# Robotic-Assisted versus Laparoscopic Surgery for Rectal Cancer: An Analysis of Clinical and Financial Outcomes from a Tertiary Referral Center

**DOI:** 10.3390/jcm13061795

**Published:** 2024-03-20

**Authors:** Jasper Max Gebhardt, Neno Werner, Andrea Stroux, Frank Förster, Ioannis Pozios, Claudia Seifarth, Christian Schineis, Benjamin Weixler, Katharina Beyer, Johannes Christian Lauscher

**Affiliations:** 1 Department of General and Visceral Surgery, Campus Benjamin Franklin—Charité University Medicine Berlin, Hindenburgdamm 30, 12203 Berlin, Germany; ja.gebhardt@uke.de (J.M.G.); ioannis.pozios@charite.de (I.P.);; 2 Department of Vascular and Endovascular Surgery, University Medical Center Hamburg-Eppendorf, Martinistraße 52, 20251 Hamburg, Germany; 3 Institute of Biometry and Clinical Epidemiology, Campus Mitte—Charité University Medicine Berlin, Charitéplatz 1, 10117 Berlin, Germany; 4 Corporate Controlling Department, Campus Mitte—Charité University Medicine Berlin, Charitéplatz 2, 10117 Berlin, Germany

**Keywords:** colorectal surgery, inpatient costs, laparoscopic surgery, surgery costs, rectal cancer, robotic-assisted surgery, total mesorectal excision

## Abstract

**Background**: The popularity of robotic-assisted surgery for rectal cancer is increasing, but its superiority over the laparoscopic approach regarding safety, efficacy, and costs has not been well established. **Methods**: A retrospective single-center study was conducted comparing consecutively performed robotic-assisted and laparoscopic surgeries for rectal cancer between 1 January 2016 and 31 September 2021. In total, 125 adult patients with sporadic rectal adenocarcinoma (distal extent ≤ 15 cm from the anal verge) underwent surgery where 66 were operated on robotically and 59 laparoscopically. **Results**: Severe postoperative complications occurred less frequently with robotic-assisted compared with laparoscopic surgery, as indicated by Clavien–Dindo classification grades 3b–5 (13.6% vs. 30.5%, *p* = 0.029). Multiple logistic regression analyses after backward selection revealed that robotic-assisted surgery was associated with a lower rate of total (Clavien–Dindo grades 1–5) (OR = 0.355; 95% CI 0.156–0.808; *p* = 0.014) and severe postoperative complications (Clavien–Dindo grades 3b–5) (OR = 0.243; 95% CI 0.088–0.643; *p* = 0.005). Total inpatient costs (median EUR 17.663 [IQR EUR 10.151] vs. median EUR 14.089 [IQR EUR 12.629]; *p* = 0.018) and surgery costs (median EUR 10.156 [IQR EUR 3.551] vs. median EUR 7.468 [IQR EUR 4.074]; *p* < 0.0001) were higher for robotic-assisted surgery, resulting in reduced total inpatient profits (median EUR −3.196 [IQR EUR 9.101] vs. median EUR 232 [IQR EUR 6.304]; *p* = 0.004). **Conclusions**: In our study, robotic-assisted surgery for rectal cancer resulted in less severe and fewer total postoperative complications. Still, it was associated with higher surgery and inpatient costs. With increasing experience, the operative time may be reduced, and the postoperative recovery may be further accelerated, leading to reduced surgery and total inpatient costs.

## 1. Introduction

Colorectal cancer is the third most prevalent cancer globally, with almost two million new cases and one million deaths per year [1]. Rectal cancer represents 28% to 35% of all colorectal cancer cases [2]. Radical resection by sphincter-preserving rectal resection with total mesorectal excision is the mainstay of treatment. Various techniques are used, including anterior resection, low anterior resection, intersphincteric resection, or abdominoperineal excision [2]. The surgical approach has developed from traditional open surgery to less invasive techniques, such as laparoscopic and robotic-assisted surgery. The laparoscopic approach reduces surgical trauma and allows for faster postoperative recovery without compromising oncologic outcomes [3,4].

Nonetheless, performing laparoscopic total mesorectal excision (TME) for rectal cancer requires a meticulous surgical technique and offers little margin for error [5]. The long, straight instruments used in laparoscopic surgery have inherent limitations in TME, such as loss of dexterity, uncontrolled assistant traction, limited range of motion, and unstable camera movement [5]. As a result, laparoscopic procedures for rectal cancer demonstrate an intraoperative conversion rate of 9–16%, increasing postoperative morbidities [6,7].

Robotic-assisted surgery aims to overcome these limitations by improving surgeon maneuverability and visibility in constrained spaces such as the lower pelvis [8]. This allows for more accurate TME, improving surgical performance and reducing the physical strain on the surgeon [9,10]. The theoretical advantages of the robotic approach imply that superior results can be achieved with robotic-assisted TME than with laparoscopic TME, especially in mid-to-low-lying rectal cancer. Nonetheless, studies comparing the surgical quality of robotic-assisted surgery for rectal cancer are required before determining its potential supremacy over the laparoscopic technique.

Hence, this study presents our initial series of consecutive patients who underwent robotic-assisted surgery for rectal cancer. We hypothesized that robotic-assisted surgery would be associated with a lower conversion rate to open surgery, lower 30-day postoperative complication rate, shorter length of hospital stay (LOS), and better quality of TME as rated by pathologists. We conducted an in-hospital cost analysis to compare the costs, revenues, and profits of both techniques in the operating room and on the ward.

## 2. Materials and Methods

### 2.1. Patient Cohort

The Department of General and Visceral Surgery at Charité Berlin—Campus Benjamin Franklin conducted a retrospective, comparative study. Our department is a specialized center for laparoscopic colorectal surgery and acts as a referral center for colorectal surgery. Robotic-assisted surgeries for rectal cancer have been performed regularly since 2016. Using the patient database of our hospital (SAP SE, Walldorf, Baden-Württemberg, Germany) [11], we identified all adult patients (age ≥ 18 years) with primary sporadic rectal adenocarcinoma who had undergone elective robotic-assisted or laparoscopic surgery between 1 January 2016 and 31 September 2021. One hundred twenty-five consecutive patients were diagnosed with primary sporadic rectal adenocarcinoma (distal extent ≤ 15 cm from the anal verge). Patients with benign tumors and/or lesions of the rectum, rectal cancer associated with inflammatory bowel disease, familial adenomatous polyposis coli, or hereditary nonpolyposis colon cancer were excluded. No patient was excluded beyond these criteria. Robotic platform availability determined the choice of surgical procedure, whereas patient characteristics did not. All robotic-assisted surgeries were performed using the Da Vinci X Surgical System (Intuitive Surgical, Mountain View, Sunnyvale, CA, USA). The study was approved by the local ethical review committee (EA4/049/21) and complied with the 1975 Declaration of Helsinki and its subsequent amendments or comparable ethical standards [11].

Inclusion criteria:

- Age ≥ 18 years;
- Sporadic rectal adenocarcinoma (distal extent ≤ 15 cm from the anal verge);
- Robotic-assisted or laparoscopic surgery;
- Elective surgery conducted between 1 January 2016 and 31 September 2021.

Exclusion criteria:

- Age ≤ 18 years;
- Benign tumors and/or lesions of the rectum;
- Colorectal cancer associated with inflammatory bowel disease, familial adenomatous polyposis coli, hereditary nonpolyposis colon cancer;
- Open surgery;
- Non-elective surgery.

### 2.2. Surgical Interventions

All five participating surgeons were experienced in laparoscopic colorectal surgery but were in the process of learning robotic-assisted rectal surgery [11]. They finished their basic training on the robotic console in 2015, 2017, 2019, 2020, and 2021 [11].

In robotic-assisted surgery, four robotic trocars (Intuitive Surgical, Mountain View, Sunnyvale, CA, USA) were placed in an oblique configuration from the lower right quadrant to the upper left quadrant (4-1-2-3 configuration). All trocars were 8 mm robotic trocars; only trocar number three (right lower quadrant) was a 12 mm robotic trocar. A 12 mm auxiliary trocar was placed between the second and third robotic trocar, a few centimeters cephalad from both (Applied Medical, Santa Clara, CA, USA). Robotic-assisted surgery was performed with fenestrated bipolar forceps in arm 1, robotic camera in arm 2, monopolar curved scissors in arm three, and Cadiere forceps in arm 4 (Intuitive Surgical, Mountain View, Sunnyvale, CA, USA). The rectum was transected at the level of the pelvic floor using a robotic linear stapler (SureForm 45 mm green cartridge; Intuitive Surgical, Mountain View, Sunnyvale, CA, USA) or a linear cutter via a Pfannenstiel incision (TA 30; Medtronic, Dublin, Ireland) [11].

Laparoscopic low anterior resection was performed with three 5 mm trocars and one 12 mm trocar (Applied Medical, Santa Clara, CA, USA). The energy device used was either a Harmonic Scalpel/Ultracision (Johnson & Johnson, New Brunswick, NJ, USA) or a Maryland Ligasure (Medtronic, Dublin, Ireland).

A medial-to-lateral approach with high-tie ligation of the inferior mesenteric artery was performed for robotic-assisted and laparoscopic surgeries. The splenic flexure was mobilized. Anastomoses were created with circular stapling (CEEA 29 mm; Johnson & Johnson, New Brunswick, NJ, USA). Anterior resection with partial mesorectal excision was performed for rectal cancer of the upper third. Low anterior resection with total mesorectal excision was performed for rectal cancer of the middle and lower third. Perfusion of the anastomotic region of the colon and rectum was checked using indocyanine green (ICG). After creating the anastomosis, rigid rectoscopy with an air leak test was performed regularly. In the case of intersphincteric resections, a hand-sewn colo-anal anastomosis was created. Extralevator abdominoperineal excision with gluteal flap reconstruction was performed for low-lying tumors with no chance of sphincter-preserving surgery.

### 2.3. Outcome Measures

Preoperative, operative, pathologic, and 30-day postoperative outcomes were retrospectively analyzed. Preoperative data included age, American Society of Anesthesiologists (ASA) classification, sex, body mass index (BMI), number of previous abdominal surgeries, smoking status, pre-existing medical conditions (diabetes, anemia, cardiovascular disease, pulmonary disease, and renal insufficiency), the height of tumor from the anal verge (grouped as follows: 0–5 cm, 5–10 cm, and 10–15 cm), neoadjuvant radiotherapy or radiochemotherapy, and pretherapeutic clinical T stage. Operative characteristics included performed surgery, operative time, conversion rate to open surgery, type of stoma formation (temporary or permanent), and intraoperative adverse events such as hemorrhage requiring transfusion, serosal tear, or damage to organ structures. Postoperative pathologic outcomes included the number of lymph nodes harvested, p/yp T stage and p/yp N stage, final histology (adenocarcinoma, dysplasia, or complete pathological response), proximal, distal, and circumferential resection margins (CRM), CRM positivity (≤1 mm), and the quality of TME as rated by pathologists using the M.E.R.C.U.R.Y. classification [12].

The primary endpoint was the rate of 30-day postoperative complications, defined as grades 1–5 of the Clavien–Dindo classification of surgical complications [13]. Postoperative complications included surgical site infection (SSI [defined according to the definition of Centers for Disease Control and Prevention]) [14], ileus (defined by insertion of a nasogastric tube at postoperative day three or later or the need for surgical revision due to ileus), anastomotic leakage (seen on abdominopelvic imaging, during endoscopy, or intraoperatively), pelvic abscess (seen on abdominopelvic imaging), hemorrhage (defined by blood count and/or abdominopelvic imaging, requiring transfusion and/or intervention), urinary tract infection (UTI [defined by a positive urinary culture and symptoms]), pulmonary artery embolism (PAE) (defined by CT imaging), pneumonia (defined by imaging and need for antibiotic therapy), and 30-day postoperative mortality [11].

The secondary endpoints were operative time, rate of conversion to open surgery, length of hospital stay (LOS), quality of TME, and detailed in-hospital cost analysis. The study population’s calculated costs, revenues, and profits refer to our Department of General and Visceral Surgery, Charité Berlin—Campus Benjamin Franklin [11]. The calculation is based on the official guidelines of the German Institute for Remuneration in Hospitals (InEK) for the calculation of German Diagnosis Related Groups ((a)G-DRGs) [11]. Our corporate controlling department uses a tool that illustrates the cost calculation and the comparison of expenses and proceeds [11]. It calculated the inpatient costs, revenues, and profits based on the case number of each patient, including surgery costs, costs on the surgical ward, costs of diagnostic procedures, laboratory costs, and medication costs [11]. Surgery costs were predominantly referred to as cutting–suture time, instrument costs, and anesthesia time [11]. Surgery costs also included costs for revision surgery [11]. Revenues were based on the (a)G-DRGs, which represent a fixed case-based total value independent of the treatment of an individual patient [15]. In Germany, there is no additional reimbursement for robotic-assisted surgery [11]. Previously published data from our research group describe the underlying composition of the inpatient cost analysis in detail [16].

### 2.4. Statistical Analysis

For categorical parameters, absolute and relative frequencies were presented, while quantitative parameters were presented using mean, standard deviation (SD), median, and interquartile range (IQR). To analyze quantitative results, we conducted statistical group comparisons using the *t*-test for independent samples. Due to the skewed distribution of certain variables, we utilized the Mann–Whitney U test to analyze group differences in quantitative variables. For categorical outcomes, we conducted group comparisons using cross-tabulations and the chi-square test.

In addition, we performed multiple logistic regression analyses to identify potential risk factors for Clavien–Dindo total complications (grades 1–5) and Clavien–Dindo severe complications (grades 3b–5). The independent variables analyzed were included based on a *p* value of 0.05 or less in a simple regression analysis with one independent variable or on clinical suspicion. We then performed stepwise backward variable elimination with a threshold *p* > 0.1. Multivariate analysis values were expressed as odds ratio (OR), 95% confidence interval (CI), and *p* value.

*p* values of 0.05 or less were considered statistically significant. Due to the exploratory nature of the analyses, no Bonferroni correction was performed. Data analysis was performed via IBM SPSS Statistics 27 (IBM, Armonk, NY, USA).

## 3. Results

One hundred twenty-five patients underwent minimally invasive surgery, of which 66 were performed robotically and 59 laparoscopically. More patients were male (*n* = 82, 65.6%), with a mean age of 63 years (SD = 12 years). Patients in both groups had an equal number of prior abdominal surgeries, with the majority not having prior surgery (68.2% vs. 64.4%; *p* = 0.694). The two groups did not differ concerning age, sex, BMI, smoking status, diabetes, anemia, cardiovascular disease, pulmonary disease, and renal insufficiency. The two groups were balanced regarding the pretherapeutic clinical T stage, with nearly half of the tumors classified as T3 (47.0% vs. 49.2%; *p* = 0.337). Patients who underwent robotic-assisted surgery were likelier to receive neoadjuvant radiotherapy or radiochemotherapy (71.2% vs. 42.4%; *p* = 0.002). In the robotic-assisted group, there was a trend toward low-lying rectal cancer, with more than twice as many tumors five centimeters or less of the anal verge (28.8% vs. 13.6%; *p* = 0.078) (Table 1).

There were no differences in operative technique between the two groups, with low anterior resection performed most frequently (72.7% vs. 69.5%; *p* = 0.945). Robotic-assisted surgery took longer on average than laparoscopic surgery (379 ± 105 min vs. 302 ± 84 min; *p* < 0.0001). Five patients in the robotic-assisted group were converted to open surgery, compared with eleven in the laparoscopic group (7.6% vs. 18.6%; *p* = 0.106). Interestingly, fewer permanent stomas were created after robotic-assisted surgery: four compared with thirteen in the laparoscopic group (6.1% vs. 22.0%; *p* = 0.037). Intraoperative adverse events such as serosal tear (3.0% vs. 1.7%; *p* = 1.000), hemorrhage requiring transfusion (1.5% vs. 3.4%; *p* = 0.602), or the rate of damage to organ structures (robotic-assisted group—spleen or ureter; laparoscopic group—spleen, ureter, ductus deferens, or bladder twice) (3.0% vs. 8.5%; *p* = 0.253) did not differ (Table 2).

On average, sixteen lymph nodes were harvested in robotic-assisted and eighteen in laparoscopic procedures (*p* = 0.140). Distance to proximal (*p* = 0.114), distal (*p* = 0.177), and circumferential resection margins (*p* = 0.631) showed no difference between the two groups; additionally, CRM positivity was comparable (13.6% vs. 8.5%; *p* = 0.407). The pathologists rated the quality of TME as complete in 85.5% of robotic-assisted cases and 92.6% of laparoscopic cases (*p* = 0.537), while R0 resection was performed in 98.5% and 98.3% of cases, respectively (*p* = 1.000) (Table 3).

The rates of postoperative SSI (9.1% vs. 6.8%; *p* = 0.748), anastomotic leakage (18.5% vs. 15.2%; *p* = 0.619), pelvic abscess (6.1% vs. 13.6%; *p* = 0.225), hemorrhage (1.5% vs. 8.5%; *p* = 0.099), UTI (16.7% vs. 6.8%; *p* = 0.105), PAE (1.5% vs. 3.4%; *p* = 0.602), and pneumonia (3.0% vs. 3.4%; *p* = 1.000) did not differ between the robotic-assisted and the laparoscopic group. Six patients who underwent robotic-assisted surgery experienced postoperative ileus, one of whom required surgical revision, versus twelve patients in the laparoscopic group, none of whom required revision (9.1% vs. 20.3%; *p* = 0.081). In the robotic-assisted group, nine patients needed to return to the operating room: four for anastomotic leakage, four for wound revision, and one for bowel perforation. In the laparoscopic group, thirteen patients required revision: three for anastomotic leakage, three for intestinal ischemia, two for wound revision, two for intestinal perforation, two for ureteral lesion, and one for trocar hernia (13.6% vs. 22.0%; *p* = 0.246). Two patients who underwent laparoscopic surgery died postoperatively from multiple organ failure after intestinal ischemia (0.0% vs. 3.4%; *p* = 0.221). There were no differences between the two groups regarding total complications (grades 1–5) in univariate analysis (51.5% vs. 66.1%, *p* = 0.106), but robotic-assisted surgery showed a significantly lower rate of severe complications (grades 3b–5) (13.6% vs. 30.5%, *p* = 0.029). There were no differences in postoperative complications between individual surgeons performing robotic-assisted or laparoscopic surgery. The median LOS was 12.5 days (IQR 10 days) for the robotic-assisted and 11.0 days (IQR 11 days) for the laparoscopic group (*p* = 0.366) (Table 4).

Multiple logistic regression analyses after backward selection revealed that laparoscopic surgery (OR = 0.355; 95% CI 0.156–0.808; *p* = 0.014) and longer operative time (OR = 1.005; 95% CI 1.001–1.010; *p* = 0.017) were associated with a higher rate of total postoperative complications. Additionally, laparoscopic surgery (OR = 0.243; 95% CI 0.088–0.643; *p* = 0.005) and low-lying rectal cancer (OR = 0.344; 95% CI 0.004–0.704; *p* = 0.004) were associated with a higher rate of severe postoperative complications (Table 5).

Robotic-assisted surgery was associated with higher total inpatient costs than laparoscopic surgery (median EUR 17,663 [IQR EUR 10,151] vs. median EUR 14,089 [IQR EUR 12,629]; *p* = 0.018). This was due to significantly higher surgery costs for the robotic platform (EUR 10,156 [IQR EUR 3551] vs. EUR 7468 [IQR EUR 4074]; *p* < 0.0001), resulting in significantly lower total inpatient profits for the robotic approach (EUR −3196 [IQR EUR 9101] vs. EUR 232 [IQR EUR 6304]; *p* = 0.004). The two groups did not differ in terms of surgical ward costs (EUR 5802 [IQR EUR 6211] vs. EUR 6622 [IQR EUR 6733]; *p* = 0.514) (Table 6).

## 4. Discussion

Some studies have demonstrated the feasibility and safety of robotic-assisted surgery for rectal cancer [8,17] and ulcerative colitis [11]. However, results could be more consistent, and the quality of evidence is variable, slowing adoption into clinical practice [18,19].

In addition to feasibility and safety, our data demonstrate several advantages of robotic-assisted surgery for rectal cancer. Most importantly, we showed that 30-day postoperative complication rates were lower for robotic-assisted compared with laparoscopic surgery for rectal cancer, as reflected by fewer Clavien–Dindo severe complications (grades 3b–5) (13.6% vs. 30.5%). In addition, multiple logistic regression analyses revealed that robotic-assisted surgery was associated with a lower rate of total and severe postoperative complications. There were no differences in postoperative complications between individual surgeons performing robotic-assisted and laparoscopic surgery. Accordingly, the study was positive concerning the primary endpoint. A systemic review and meta-analysis by Wang et al. [20] and a multicenter, randomized, controlled, superiority trial by Feng et al. [21] also showed significantly lower severe complication rates for the robotic approach. The latter study attributed this to significantly less surgical trauma and improved postoperative recovery due to enhanced visibility and a greater range of motion within the narrow pelvis. Aside from that, multiple logistic regression analyses associated longer operative time with more Clavien–Dindo total complications and low-lying rectal cancer with more Clavien–Dindo severe complications. Both associations can be considered surrogate parameters, as it has been repeatedly shown that longer operative time [22] and low-lying rectal cancer [23] are associated with higher postoperative complication rates. As operative time may be reduced with increasing surgeon experience, complication rates could decrease.

Interestingly, there was a trend toward less postoperative ileus and a lower postoperative hemorrhage rate in the robotic group. It is possible that more careful dissection of the TME plane with the robotic platform resulted in less tissue trauma and bleeding, leading to less inflammation [24] and adhesion formation and, consequently, less postoperative ileus compared with the laparoscopic group [25]. There were no differences between robotic-assisted and laparoscopic procedures regarding other postoperative complications. Two deaths occurred in the laparoscopic group but none in the robotic group. Both patients died of multiple organ failure after revision surgery for intestinal ischemia.

We compared conversion rates between the two approaches to test the hypothesis that the robotic platform’s technical advantages should facilitate TME and avoid the need for conversion to open surgery. The conversion rate for robotic-assisted surgery was 7.6%, whereas the conversion rate for laparoscopic surgery was high at 18.6%. Although only a trend, it appears that the robotic platform, with its articulated instruments, stable camera platform, and immersive three-dimensional depth of field, allows surgeons to overcome some of the restrictions of laparoscopic rectal cancer surgery, resulting in a moderately reduced conversion rate [26,27].

In this study, the rate of temporary stoma formation was higher in the robotic group than in the laparoscopic group (89.4% vs. 72.9%), possibly because the robotic group had more patients with low-lying rectal cancer. Potentially, the robotic approach allows meticulous dissection deep to the pelvic floor with more colorectal or coloanal anastomoses and temporary ileostomy instead of abdominoperineal excision [28,29].

Consistent with other studies [17,18], robotic-assisted surgery took longer than laparoscopic surgery. Interestingly, compared with other studies [30,31], we did not demonstrate a gradual decrease in operative time as the surgeons gained more experience. Approximately 25–30 procedures per surgeon are estimated to be required before a relevant reduction in operative time is detected [32]. Only one of the participating surgeons achieved this number of robotic-assisted surgeries for rectal cancer during the study period.

Consistent with other studies [33,34], we found that robotic-assisted surgery for rectal cancer provided equivalent pathologic outcomes compared with laparoscopic surgery regarding the number of lymph nodes harvested, CRM-positivity, and quality of TME as rated by pathologists. The quality of surgery performed in our study is underlined by the high quality of TME. Consistent with other studies [17,18,19], we observed a success rate of 85.5% for robotic-assisted and 92.6% for laparoscopic surgery. However, we did not demonstrate a higher quality of TME for robotic-assisted surgery. There were more low-lying tumors in the robotic-assisted group (28.8% vs. 13.6%), with consequently higher rates of neoadjuvant radiotherapy or radiochemotherapy (71.2% vs. 42.4%), which may have complicated dissection, compromising the pathologic outcome for robotic-assisted surgery. Neoadjuvant radiotherapy or radiochemotherapy is known to cause edema and fibrosis in patients with rectal cancer, which complicates the procedure by increasing smoke production and fluid leakage, making it more challenging to separate surgical planes [35]. These effects could have negated the benefit of tumor reduction.

Like other studies, we did not demonstrate shorter LOS for patients who received robotic-assisted surgery [17,18]. The hospital stay in Germany tends to be relatively long, as patients are only discharged once they are fit and self-dependent.

To the best of our knowledge, this is one of the most comprehensive economic evaluations comparing inpatient healthcare costs of robotic-assisted and laparoscopic surgery for rectal cancer [18,36,37]. The corporate controlling team evaluated healthcare expenses based on the (a) G-DRG system [11]. Accurate estimates of costs, revenues, and profits were calculated based on each patient’s case number [11]. Our findings indicate that robotic-assisted surgery for rectal cancer is unlikely cost-saving. Surgery costs were higher for the robotic approach than for the laparoscopic approach, EUR 10,156 vs. EUR 7468. The difference was mainly due to higher costs for robotic instruments and longer operative time. However, surgical ward costs did not differ between the two techniques because of comparable LOS (EUR 5802 vs. EUR 6622). Surgical costs and surgical ward costs together constitute the total inpatient costs [11]. These were higher for robotic-assisted compared with laparoscopic surgery (median EUR 17,663 vs. median EUR 14,089). Robotic procedures do not generate additional revenues compared with laparoscopic procedures in the (a)G-DRG system [11]. These combined aspects resulted in lower total inpatient profits for robotic-assisted surgery (EUR −3196 vs. EUR 232).

When considering robotic-assisted surgery, it is essential to consider acquisition and maintenance costs, operational life, and the total utilization of the robotic platform per year—a detailed health economic analysis by Jayne et al. [18], based on 2017 data from Intuitive Surgical (Mountain View, Sunnyvale, CA, USA), states that “the net benefits (excluding fixed costs) of each robotic-assisted operation included in a set of cost-effective procedures must be positive, and the entire set of cost-effective procedures must have an average net benefit of at least EUR 1491 (USD 1611). On average, all robotic-assisted operations combined must exceed this figure, with each operation making at least some positive contribution.” Based on data from our retrospective study, robotic-assisted surgery for rectal cancer was associated with a median increase in total inpatient costs of EUR 3574 (USD 3931) per case. It should be noted that all surgeons had ample experience in laparoscopic colorectal surgery [11]. On the other hand, they were in the process of learning robotic-assisted colorectal surgery [11]. This may have confounded our results. With increasing experience, the postoperative 30-day complication rate and LOS could further decrease, positively impacting costs [18,36,37].

It is rational to advance the platform’s technology as its potential for improvement can lead to further clinical benefits. The prospects for further advances in robotic technology exceed those of laparoscopy [38]. As new competitors and more advanced robotic platforms enter the market, the acquisition, maintenance, and instrument costs will probably decrease [39]. Given the circumstances, robotic platforms are likely to become a standard component of colorectal surgery, particularly for complex low anterior resections.

### Limitations

This study has several limitations. First, it is a monocentric retrospective comparative study from a prospectively maintained database, which limits the analysis of postoperative outcomes. In future studies, data from several centers will be analyzed to increase the generalizability of the results. Second, our department is a specialized center for laparoscopic colorectal surgery and functions as a referral center for colorectal surgery. Therefore, our results may not be directly applicable to other institutions due to differences in patient population, preoperative setting, and operative steps. Third, despite the mandatory minimum experience of participating surgeons, the operations were performed by surgeons with ample experience in laparoscopic colorectal surgery but still in the process of learning robotic-assisted rectal cancer surgery. Fourth, the number of patients is still relatively small but will grow as experience with robotic-assisted surgery for rectal cancer increases. Because of the relatively small number of patients, the study may not have been sufficiently powered to detect significant differences. Fifth, patients in the robotic group were more likely to receive neoadjuvant radiotherapy or radiochemotherapy, partly because of low-lying rectal cancer. This may have confounded the results regarding postoperative complications, conversion rate, hospital stay, and costs. Considering these limitations, our data suggest that robotic-assisted surgery for rectal cancer is feasible and has at least comparable short-term outcomes to the laparoscopic approach.

The study’s strength is the detailed assessment of specific clinical, pathologic, and financial outcomes for robotic-assisted and laparoscopic surgery for rectal cancer. To the best of our knowledge, this is one of the most comprehensive economic evaluations comparing inpatient healthcare costs of robotic-assisted surgery and laparoscopic surgery for rectal cancer. All 125 consecutive patients were diagnosed with primary sporadic rectal adenocarcinoma. Hence, our cohort was homogenous and comparable; the two groups were balanced. All robotic-assisted procedures were conducted by surgeons who had already achieved expert status in laparoscopic colorectal surgery. They adhered to well-established surgical training, proctoring, and protocol while utilizing the Da Vinci X Surgical System.

## 5. Conclusions

Robotic-assisted surgery is a safe and effective technique for patients with rectal cancer. Data from this study suggest that postoperative complication rates may be lower with robotic-assisted surgery compared with laparoscopic surgery, likely due to less surgical trauma and more delicate tissue handling. Total inpatient and surgery costs were higher for robotic-assisted surgery, reducing total inpatient profits. As experience increases and after overcoming a learning curve, operative time may be reduced, and postoperative recovery may be accelerated.

## Figures and Tables

**Table 1:** Patient baseline characteristics.

Variable	Robotic-Assisted Surgery (*n* = 66)	Laparoscopic Surgery (*n* = 59)	*p* Value
Age, Mean ± SD, years	63 ± 12	62 ± 11	0.782
ASA classification, No. (%)			0.457
1—Normal healthy patient	7 (10.6)	10 (17.5)	
2—Patient with mild systemic disease	44 (66.7)	33 (57.9)	
3—Patient with severe systemic disease	15 (22.7)	13 (22.8)	
4—Patient with severe systemic disease that is a constant threat to life	0 (0.0)	1 (1.8)	
Sex, No. (%)			0.259
Female	26 (39.4)	17 (28.8)	
Male	40 (60.6)	42 (71.2)	
BMI, Mean ± SD, kg/m^2^	25.2 ± 4.3	24.8 ± 4.1	0.658
Previous abdominal surgeries, No. (%)			0.694
None	45 (68.2)	38 (64.4)	
1	16 (24.2)	18 (30.5)	
2–3	5 (7.6)	3 (5.1)	
Current smoking, No. (%)	6 (9.1)	10 (16.9)	0.283
Pre-existing medical condition, No. (%)			
Diabetes	5 (7.6)	3 (5.1)	0.721
Anemia	1 (1.5)	2 (3.4)	0.602
Cardiovascular disease	5 (7.6)	6 (10.2)	0.755
Pulmonary disease	4 (6.1)	3 (5.1)	1.000
Renal insufficiency	2 (3.0)	3 (5.1)	0.666
Height of tumor from anal verge, cm, No. (%)			0.078
11–15	15 (22.7)	21 (35.6)	
6–10	32 (48.5)	30 (50.8)	
0–5	19 (28.8)	8 (13.6)	
Neoadjuvant radiotherapy or radiochemotherapy, No. (%)	47 (71.2)	25 (42.4)	0.002 *
Pretherapeutic clinical T-stage, No. (%)			0.337
T0	9 (13.6)	3 (5.1)	
T1	1 (1.5)	4 (6.8)	
T2	14 (21.2)	12 (20.3)	
T3	31 (47.0)	29 (49.2)	
T4	10 (15.2)	10 (16.9)	
Missing data	1 (1.5)	1 (1.7)	

Data are presented as *n* (%) or mean ± SD; bold characters indicate significant values, * *p* ≤ 0.05. Abbreviations: ASA, American Society of Anesthesiologists; BMI, body mass index (calculated as weight in kilograms divided by height in meters squared).

**Table 2:** Operative data.

Variable	Robotic-Assisted Surgery (*n* = 66)	Laparoscopic Surgery (*n* = 59)	*p* Value
Performed operation, No. (%)			0.945
Anterior resection	9 (13.6)	7 (11.9)	
Low anterior resection	48 (72.7)	41 (69.5)	
Intersphincteric resection	3 (4.5)	3 (5.1)	
Proctocolectomy	1 (1.5)	1 (1.7)	
Abdominoperineal excision	5 (7.6)	7 (11.9)	
Operative time, Mean ± SD, min	379 ± 105	302 ± 84	<0.0001 *
Conversion to open surgery, No. (%)	5 (7.6)	11 (18.6)	0.106
Stoma formation, No. (%)			0.037 *
Temporary	59 (89.4)	43 (72.9)	
Permanent	4 (6.1)	13 (22.0)	
Intraoperative adverse event, No. (%)			
Serosal tear	2 (3.0)	1 (1.7)	1.000
Hemorrhage requiring transfusion	1 (1.5)	2 (3.4)	0.602
Damage to organ structures	2 (3.0)	5 (8.5)	0.253

Data are presented as *n* (%) or mean ± SD; bold characters indicate significant values, * *p* ≤ 0.05. Abbreviations: SD, standard deviation.

**Table 3:** Postoperative pathological outcomes.

Variable	Robotic-Assisted Surgery (*n* = 66)	Laparoscopic Surgery (*n* = 59)	*p* Value
Lymph node yield, Mean ± SD, No.	16.3 ± 5.4	18.0 ± 7.6	0.140
*p*/ypT classification, No. (%)			0.420
T0	10 (15.2)	8 (13.6)	
T1	3 (4.5)	2 (3.4)	
T2	16 (24.2)	23 (39.0)	
T3	34 (51.5)	24 (40.7)	
T4a	3 (4.5)	1 (1.7)	
T4b	0 (0.0)	1 (1.7)	
p/ypN classification, No. (%)			0.990
N0	42 (63.6)	37 (62.7)	
N1a	5 (7.6)	4 (6.8)	
N1b	6 (9.1)	6 (10.2)	
N1c	5 (7.6)	3 (5.1)	
N2a	4 (6.1)	5 (8.5)	
N2b	4 (6.1)	4 (6.8)	
Final histology, No. (%)			1.000
Adenocarcinoma	54 (81.8)	49 (83.1)	
Dysplasia	4 (6.1)	3 (5.1)	
Complete response	8 (12.1)	7 (11.9)	
Proximal resection margin, cm, median (IQR)	20.5 (9.8)	22.0 (11.0)	0.114
Distal resection margin, cm, median (IQR)	2.2 (3.0)	3.0 (3.8)	0.177
Circumferential resection margin, cm, median (IQR)	1.5 (1.8)	1.6 (1.4)	0.631
Circumferential resection margin positive (≤1 mm), No. (%)	9 (13.6)	5 (8.5)	0.407
R0 resection, No. (%)	65 (98.5)	58 (98.3)	1.000
Quality of TME as rated by pathologist, No. (%)			0.537
Complete	53/62 (85.5)	50/54 (92.6)	
Nearly complete	8/62 (12.9)	3/54 (5.6)	
Incomplete	1/62 (1.6)	1/54 (1.9)	

Data are presented as *n* (%) or mean ± SD or median or interquartile range. Abbreviations: SD, standard deviation; IQR, interquartile range; TME, total mesorectal excision.

**Table 4:** Postoperative complications within 30 days.

Variable	Robotic-Assisted Surgery (*n* = 66)	Laparoscopic Surgery (*n* = 59)	*p* Value
Postoperative complications, No. (%)			
Surgical site infection	6 (9.1)	4 (6.8)	0.748
Postoperative ileus	6 (9.1)	12 (20.3)	0.081
Anastomotic leakage	12/62 (18.5)	7/46 (15.2)	0.619
Pelvic abscess	4 (6.1)	8 (13.6)	0.225
Hemorrhage	1 (1.5)	5 (8.5)	0.099
Urinary tract infection	11 (16.7)	4 (6.8)	0.105
Pulmonary artery embolism	1 (1.5)	2 (3.4)	0.602
Pneumonia	2 (3.0)	2 (3.4)	1.000
Return to operating room, No. (%)	9 (13.6)	13 (22.0)	0.246
Clavien–Dindo classification, No. (%)			
Total complications, grade I–V	34 (51.5)	39 (66.1)	0.106
Severe complications, grade IIIb–V	9 (13.6)	18 (30.5)	0.029 *
30-day mortality, No. (%)	0 (0.0)	2 (3.4)	0.221
Length of stay (days), Median (IQR)	12.5 (10.0)	11.0 (11.0)	0.366

Data are presented as *n* (%) or median or interquartile range; bold characters indicate significant values, * *p* ≤ 0.05. Abbreviation: IQR, interquartile range.

**Table 5:** Multiple logistic regression analysis of potential risk factors for Clavien–Dindo total complications and Clavien–Dindo severe complications.

Dependent Variable	Variable	OR	95% CI		*p* Value
			Lower	Upper	
Clavien–Dindo total complications	Rob. vs. Lap.	0.355	0.808	0.156	0.014 *
	Operative time	1.005	1.001	1.010	0.017 *
Clavien–Dindo severe complications	Rob. vs. Lap.	0.243	0.643	0.088	0.005 *
	Height of tumor from anal verge	0.344	0.168	0.704	0.004 *

The analyzed independent variables were included due to a *p* ≤ 0.05 on simple regression analysis with one independent variable or on clinical suspicion. We then performed stepwise backward variable elimination with threshold *p* > 0.1; reference categories: Rob. vs. Lap, laparoscopic surgery; the height of tumor from anal verge, 0–5 cm; data are presented as odds ratio and confidence intervals; bold characters indicate significant values, * *p* ≤ 0.05. Abbreviations: CI, confidence interval; Lap., laparoscopic; OR, odds ratio; Rob., robotic.

**Table 6:** In-hospital cost analysis.

Category	Robotic-Assisted Surgery (*n* = 66)	Laparoscopic Surgery (*n* = 59)	*p* Value
Total inpatient costs			0.018 *
Median	EUR 17,663 (USD 19,429)	EUR 14,089 (USD 15,498)	
IQR	EUR 10,151 (USD 11,166)	EUR 12,629 (USD 13,892)	
Q25	EUR 14,093 (USD 15,502)	EUR 12,542 (USD 13,796)	
Q75	EUR 24,244 (USD 26,668)	EUR 25,171 (USD 27,688)	
Total inpatient revenues			0.619
Median	EUR 14,883 (USD 16,371)	EUR 14,883 (USD 16,371)	
IQR	EUR 2140 (USD 2354)	EUR 3260 (USD 3586)	
Q25	EUR 13,806 (USD 15,187)	EUR 12,959 (USD 14,255)	
Q75	EUR 15,946 (USD 17,541)	EUR 16,219 (USD 17,841)	
Total inpatient profits			0.004 *
Median	EUR −3196 (USD −3516)	EUR 232 (USD 255)	
IQR	EUR 9101 (USD 10,011)	EUR 6304 (USD 6934)	
Q25	EUR −8749 (USD −9623)	EUR −4010 (USD −4411)	
Q75	EUR 352 (USD 387)	EUR 2294 (USD 2523)	
Surgery costs			<0.0001 *
Median	EUR 10,156 (USD 11,172)	EUR 7468 (USD 8215)	
IQR	EUR 3551 (USD 3906)	EUR 4074 (USD 4482)	
Q25	EUR 8706 (USD 9577)	EUR 5291 (USD 5820)	
Q75	EUR 12,257 (USD 13,483)	EUR 9365 (USD 10,302)	
Surgery revenues			0.167
Median	EUR 6459 (USD 7105)	EUR 6276 (USD 6904)	
IQR	EUR 1379 (USD 1516)	EUR 1577 (USD 1734)	
Q25	EUR 5592 (USD 6151)	EUR 5168 (USD 5685)	
Q75	EUR 6970 (USD 7667)	EUR 6745 (USD 7419)	
Surgery profits			<0.0001 *
Median	EUR −3692 (USD −4061)	EUR −996 (USD −1095)	
IQR	EUR 4231 (USD 4654)	EUR 2910 (USD 3201)	
Q25	EUR −6462 (USD −7108)	EUR −2461 (USD −2707)	
Q75	EUR −2231 (USD −2454)	EUR 449 (USD 494)	
Surgical ward costs			0.514
Median	EUR 5802 (USD 6382)	EUR 6622 (USD 7284)	
IQR	EUR 6211 (USD 6832)	EUR 6733 (USD 7406)	
Q25	EUR 4266 (USD 4692)	EUR 4221 (USD 4643)	
Q75	EUR 10,476 (USD 11,524)	EUR 10,954 (USD 12,049)	
Surgical ward revenues			0.917
Median	EUR 7097 (USD 7807)	EUR 6774 (USD 7451)	
IQR	EUR 1437 (USD 1580)	EUR 1900 (USD 2090)	
Q25	EUR 6333 (USD 6966)	EUR 6367 (USD 7004)	
Q75	EUR 7769 (USD 8546)	EUR 8267 (USD 9094)	
Surgical ward profits			0.477
Median	EUR 831 (USD 914)	EUR 230 (USD 253)	
IQR	EUR 4457 (USD 4902)	EUR 4806 (USD 5286)	
Q25	EUR −2159 (USD −2375)	EUR −2624 (USD −2886)	
Q75	EUR 2298 (USD 2527)	EUR 2182 (USD 2400)	

Data are presented as median or quartile or interquartile in EUR and USD; bold characters indicate significant values, * *p* ≤ 0.05; exchange rate from EUR in USD based on 5 October 2023. Abbreviations: EUR, Euro; IQR, interquartile range; Q, quartile; USD, United States Dollar.

## Data Availability

Publicly available datasets were analyzed in this study. These data can be found at https://www.synapse.org/#!Synapse:syn53277960/wiki/625734; accessed on 15 January 2024 and added on 12 March 2024.
